# Inferring Ecological Processes from Taxonomic, Phylogenetic and Functional Trait β-Diversity

**DOI:** 10.1371/journal.pone.0020906

**Published:** 2011-06-17

**Authors:** James C. Stegen, Allen H. Hurlbert

**Affiliations:** Department of Biology, University of North Carolina, Chapel Hill, North Carolina, United States of America; University of Copenhagen, Denmark

## Abstract

Understanding the influences of dispersal limitation and environmental filtering on the structure of ecological communities is a major challenge in ecology. Insight may be gained by combining phylogenetic, functional and taxonomic data to characterize spatial turnover in community structure (β-diversity). We develop a framework that allows rigorous inference of the strengths of dispersal limitation and environmental filtering by combining these three types of β-diversity. Our framework provides model-generated expectations for patterns of taxonomic, phylogenetic and functional β-diversity across biologically relevant combinations of dispersal limitation and environmental filtering. After developing the framework we compared the model-generated expectations to the commonly used “intuitive” expectation that the variance explained by the environment or by space will, respectively, increase monotonically with the strength of environmental filtering or dispersal limitation. The model-generated expectations strongly departed from these intuitive expectations: the variance explained by the environment or by space was often a unimodal function of the strength of environmental filtering or dispersal limitation, respectively. Therefore, although it is commonly done in the literature, one cannot assume that the strength of an underlying process is a monotonic function of explained variance. To infer the strength of underlying processes, one must instead compare explained variances to model-generated expectations. Our framework provides these expectations. We show that by combining the three types of β-diversity with model-generated expectations our framework is able to provide rigorous inferences of the relative and absolute strengths of dispersal limitation and environmental filtering. Phylogenetic, functional and taxonomic β-diversity can therefore be used simultaneously to infer processes by comparing their empirical patterns to the expectations generated by frameworks similar to the one developed here.

## Introduction

Understanding the processes that govern the assembly of local communities from regional species pools is a fundamental goal of ecological research, and both stochastic and deterministic factors are commonly thought to be important. Stochastic factors involve chance or historical contingency, and include processes such as dispersal limitation and ecological drift through random birth/death events [1], [2]. Deterministic factors include niche-based processes such as environmental filtering, competition and predation [3], [4]. It is clear that both stochastic and deterministic processes are at work simultaneously in most communities [5], [6], [7], [8], and recent work has focused on evaluating the relative importance of these two sets of processes and on elucidating the factors that may shift that relative importance [9], [10], [11], [12], [13], [14], [15].

One approach for inferring the relative influences of stochastic and deterministic processes is to examine spatial turnover in community structure by relating the amount of turnover (β-diversity) to variation in spatial distance and the abiotic environment [16], [17], [18], [19]. This approach has been widely employed in studies of taxonomic β-diversity by characterizing communities in terms of lists of species names with or without information on relative abundances (reviewed in [20], [21]). Several authors have recently proposed extending this species-based approach by including functional and phylogenetic information which should permit inferences that are more directly tied to ecological and evolutionary processes [22], [23].

The inclusion of phylogenetic and functional information may provide greater insight into the processes governing community structure, but the utility of these additional layers of information has not been critically evaluated. An increasing number of studies have attempted to characterize patterns of phylogenetic and functional β-diversity [24], [25], [26], [27], and with the increase in species-level phylogenetic and functional trait data this trend will continue. However, several impediments exist with respect to using results from such studies to link β-diversity patterns to the processes of community assembly. For example, there are a number of metrics developed for phylogenetic or functional β-diversity, potentially making comparisons among studies difficult [20], [21], [28], yet we lack an understanding of how phylogenetic and functional β-diversity metrics relate to each other. More importantly, the field currently lacks an explicit theoretical framework that can guide interpretation when studies simultaneously examine empirical patterns of taxonomic, phylogenetic and functional β-diversity. In the absence of such a framework, it will be difficult to move beyond pattern description, especially when each type of β-diversity responds in a different way to the same set of underlying processes.

Researchers have generally assumed that the variance in β-diversity explained by the environment increases monotonically with the strength of environmental filtering (e.g., [7], [9], [11], [25], [29], [30], [31], [32], [33]). However, Smith and Lundholm [34] recently showed that this ‘intuitive’ expectation is not valid. When community assembly is simulated under known processes, the variance in β-diversity explained by the environment is often a unimodal or other non-monotonic function of the strength of environmental filtering. Variance partitioning results cannot therefore be used to directly infer the strength of underlying processes.

One potential solution to this issue is to interpret empirical variance partitioning results with respect to *a priori* expectations derived from community assembly simulation models. Interpreting the results of variance partitioning in the context of appropriate assembly models is roughly analogous to and as important as interpreting traditional community assembly rules (e.g. Diamond's checkerboards) with respect to appropriate null models (e.g., [35], [36], [37]). However, the simulation-based approach is distinct from a traditional null model in that there is no single ‘null’ expectation, but rather a suite of expectations generated under different sets of processes. Sets of expectations are then ‘competed’ against each other based on their fit to empirical patterns. This is conceptually similar to the ‘pattern oriented modeling’ approach discussed in Grimm et al. [38], and goes beyond the binary test provided by most null models (i.e., is community structure random or not?) by allowing inferences regarding the relative and absolute magnitudes of community assembly processes (see also [39], [40], [41]).

Here we have two broad goals. The first is to develop a practical framework that can be used by empiricists that want to infer processes of community assembly by simultaneously examining empirical patterns of taxonomic, phylogenetic and/or functional β-diversity. The inclusion of phylogenetic and functional data in this framework requires the development of substantially more sophisticated community assembly models than previously developed. As such, we provide guidance so that other researchers can use our framework to first generate expected patterns of β-diversity across relevant combinations of community assembly processes and then compare empirical patterns to those expectations. Doing so will allow inference of the relative *and* absolute strengths of processes that govern empirical patterns of community structure. Our second goal is to provide a framework that can be added to or modified by theoreticians eager to explore additional assumptions and processes relevant to community assembly.

In working towards these goals we (*i*) describe a set of taxonomic, phylogenetic and functional β-diversity metrics; (*ii*) develop the analytical and simulation models that comprise our framework; (*iii*) use the framework to generate expectations under an empirically realistic scenario; (*iv*) compare model-generated expectations for taxonomic, phylogenetic and functional β-diversity to intuition-based expectations commonly employed in the literature; and (*v*) test the utility of the framework to provide inferences of underlying processes by combining taxonomic, phylogenetic and functional β-diversity.

## Methods

### β-Diversity Metrics

A wide range of metrics exist for quantifying β-diversity [20], [21], [28], [42]. Here we examine a subset that quantify the degree of taxonomic, phylogenetic, or functional dissimilarity between communities. For phylogenetic and functional data we investigate (*i*) mean phylogenetic or functional pairwise distances (*PW*) (Eq. 1), a between-community version of the net relatedness index of Webb ([43], see also the taxonomic distinctness metric reviewed in [44]); (*ii*) mean nearest neighbor distance (*NN*) (Eq. 2) in either phylogenetic or functional trait space [45], [46], [47], [48], a between-community metric similar to the nearest taxon index of Webb [43]; and (*iii*) a version of Sørensen's metric (*SOR*) (Eq. 3) that accounts for shared and unique branch lengths in a phylogeny or functional trait dendrogram [22], [49]. For taxonomic data we examined the classical *SOR* metric and its abundance-weighted version, Bray-Curtis (*BC*) (Eq. 4).

Equations (1) and (2) provide general forms of *PW* and *NN* where both are based on differences among individuals across communities. That is, both are weighted by the relative abundances of species within communities and by the relative community abundance (i.e. number of individuals of all species in a community relative to the total number of individuals across all communities).
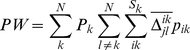
(1)

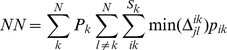
(2)Relative abundance of species *i* in site *k* and the relative community abundance of site *k* are given by *p_ik_* and *P_k_*, respectively. There are *S_k_* species in community *k*, *N* communities being compared and 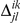 is a vector of distances (functional or phylogenetic) between a single species *i* in *k* and all species *j* in site *l*, and 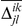 is the mean of that vector.

The phylogenetic or functional trait *SOR* metric is defined as
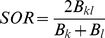
(3), where *B_kl_* is the shared branch lengths between sites *k* and *l*, and *B_k_* and *B_l_* are the total branch lengths for site *k* and *l*, respectively. Branch lengths are from either a phylogeny or a functional trait dendrogram. It is important to note that functional *PW* and *NN* do not require the use of a trait dendrogram, although a dendrogram can be used if desired. In addition, *SOR* does not incorporate species or community relative abundances.

The *BC* metric, which uses species relative abundances to quantify taxonomic turnover between two communities, is given by
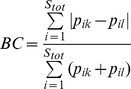
(4), where *S_tot_* is the total number of species in the two communities. If species' relative abundances are converted into presence-absence values (0 or 1), Eq. (4) collapses to one minus the taxonomic *SOR* metric, which is directly analogous to *SOR* as presented in Eq. (3) [22], [49].

### Generating Expectations: A General Overview

Patterns of β-diversity in a world in which all species were extremely dispersal-limited would look quite different from patterns in a world in which all species could reliably colonize distant locations. We can imagine a spectrum of possibilities between those two extremes, and across this spectrum β-diversity patterns (e.g. their shape or strength) will vary in some predictable fashion. The details of those patterns will depend upon the particular assumptions we make about how dispersal-limitation affects community assembly. However, given some reasonable set of assumptions and an empirical β-diversity pattern, we can begin to zero in on where the empirical system lies along the dispersal limitation spectrum. One can imagine a parallel situation for identifying the position of an empirical system along a spectrum of environmental filtering. Below we describe an approach for generating sets of local communities and expected β-diversity patterns based on the strengths of dispersal limitation and environmental filtering.

**Figure 1:**
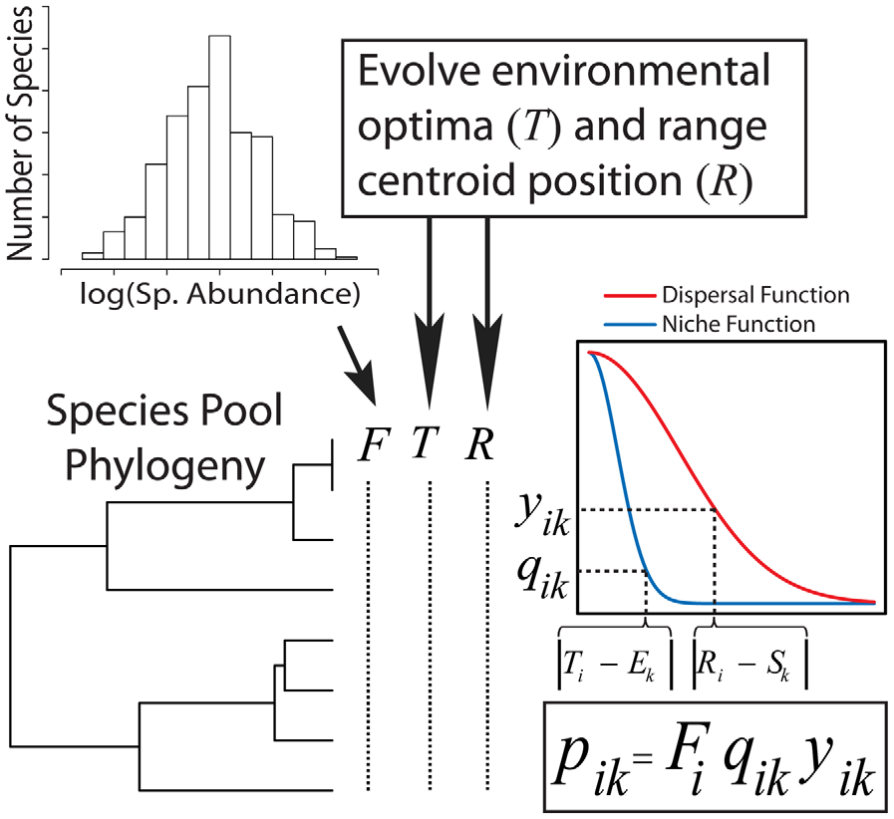
Simulation procedure for a simplified scenario with one spatial dimension and one environmental variable. Note that capitalized variables indicate vectors. After defining the strengths of niche breadth (*n*) and dispersal breadth (*d*) the regional species pool phylogeny was generated (shown with 8 species for simplicity), species' global relative abundances (*F*) were randomly assigned from a lognormal abundance distribution, and species' environmental optima (*T*) along the environmental axis were evolved while tracking species' range centroids (*R*) along the spatial dimension. Environmental conditions (*E*) and spatial positions (*S*) were then defined for twenty local sites. To assemble a community at site *k* the environmental condition (*E_k_*) and spatial position (*S_k_*) of site *k* were compared to the environmental optima (*T_i_*) and range centroid (*R_i_*), respectively, for each species *i*. From these environmental and spatial differences probabilities were found from Gaussian distributions (blue and red curves), the variances of which were *n* and *d*, respectively. A probability of incidence for species *i* at site *k* (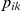) was then found as the product of the global relative abundance (*F_i_*) and probabilities based on environmental (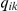) and spatial (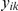) distances for species *i*. To generate the vector of relative abundances for site *k* the regional species pool was then sampled 10,000 times with replacement, where 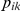 gave the probability of choosing species *i*.

**Figure 2:**
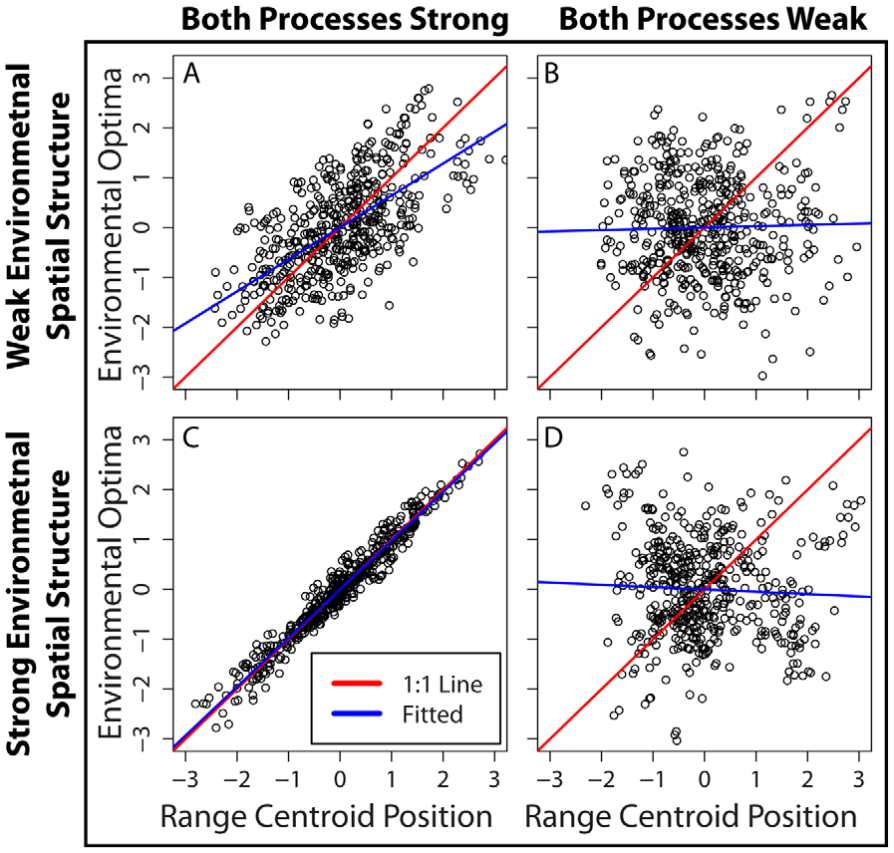
Effects of assembly processes on the relationship between environmental optima and range centroids. The strengths of environmental filtering, dispersal limitation, and environmental spatial structure constrain the evolutionary-time-scale relationship between species' intrinsic environmental optima (functional trait value) and the spatial position where their abundance is maximized (the range centroid). Simulation output when both processes are strong (*n* = *d* = 0.0001; panels A,C) or weak (*n* = *d* = 10; panels B,D). The environment has either weak (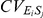
≈0.3; panels A,B) or strong (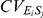
≈0.95; panels C,D) spatial structure. All axes are normalized as standard normal deviates, with mean zero and standard deviation of one. Solid red lines represent the one to one line and solid blue lines are linear regressions. (A) When both processes are strong but there is little environmental spatial structure, a moderately tight relationship emerges between species' trait values and the positions of their range centroids. (C) Increasing the degree of environmental spatial structure leads to a much tighter, one to one relationship. (B,D) Irrespective of how spatially structured the environment is, when both processes are weak there is no relationship between species' trait values and the positions of their range centroid.

Any model generating expected patterns should be constrained with system-specific attributes so that expectations are relevant to the empirical system of interest. As such, we develop our model so that it can use empirical attributes as inputs. These empirical attributes may include, but are not limited to, (*i*) the regional species pool phylogeny; (*ii*) the degree of phylogenetic trait conservatism; (*iii*) the spatial distribution of local communities; (*iv*) the degree to which an environmental variable (e.g. temperature) is correlated to a given spatial dimension (e.g. latitude); and (*v*) the number of individuals or species in local communities (see also Table 1). To generate expected patterns of taxonomic, functional and phylogenetic β-diversity our framework uses these inputs to combine an analytical model with simulations in six general steps (Fig. 1):

1. Define the range of strengths of environmental filtering and dispersal limitation to be examined. Each model run generates expected β-diversity patterns for a unique combination of process strengths.
2. Define local sites with respect to their geographic coordinates and environmental conditions. The strength of the environment-space correlation across these sites will affect the ability of variance partitioning to uniquely ascribe variance to space or the environment. As such, it is important that the model be constrained to the environment-space correlation of the empirical system.
3. Evolve species environmental optima [traits] across the regional species pool phylogeny. At the same time that traits are evolving, species geographic ranges are allowed to move through space. For simplicity the spatial position of a species range is summarized by its center of abundance, referred to here as the ‘range centroid.’ For any given species, trait evolution and changes in the position of its range centroid are not independent. The covariance between them is determined by the strength of dispersal limitation, the strength of environmental filtering, and the degree of environmental spatial structure (see Fig. 2 and the following section for details).
4. Assign species relative abundances throughout the regional species pool. This is done by randomly drawing from a defined species abundance distribution, which can be constrained to match the empirical distribution.
5. Community assembly occurs through the probabilistic assignment of individuals to local sites based on species' relative abundances in the regional species pool, the spatial proximity of a species range centroid to a given site, the strength of dispersal limitation, the match between species' environmental optima and the environment of a site, and the strength of environmental filtering. For example, if dispersal-limitation is strong and environmental filtering is weak, a species' probability of inclusion depends primarily on the proximity of its center of abundance (range centroid) to the local site. An assumption here is that species can disperse from any location within their range, but abundance declines towards the range edge so that distance of a site from the range centroid provides a reasonable summary of the probability that a species is found in that site. If community assembly is also governed by strong environmental filtering, the probability of inclusion also depends upon the difference between the species' environmental optima and the environmental conditions at the site.
6. Quantify β-diversity for all pairwise community comparisons using selected β-diversity metrics. In turn, variation in each β-diversity metric is partitioned into components explained by space only, environment only, spatially structured environment, and residuals [50]. We take this approach because the relative influences of dispersal limitation and environmental filtering are often inferred in empirical studies through variance partitioning of β-diversity. As noted above, the assumption in these empirical studies is that the variance explained by the environment, for example, increases with the strength of environmental filtering. Using variance partitioning on the model-generated expectations provides the opportunity to directly test this assumption.

**Table 1:** Major assumptions and tools used in the theoretical framework developed here.

Current Framework Assumptions and Tools	Constrain Empirically?	Alternative Formulation
Environment-Space correlation with R^2^ = 0.5	Yes	Empirical correlation
Local sites randomly located in space	Yes	Spatial clustering of sites
All local sites have equal community abundance	Yes	Allow abundances to vary
Local species richness not constrained	Yes	Limit richness, not abundance
Randomly assign species global abundances	Yes	Closely related species with similar abundances
Species abundance distribution is lognormal	Yes	Uniform distribution
Regional species pool of 500 species	Yes	Empirical regional richness
Simulated phylogeny with no extinction	Yes	Empirical phylogeny
Traits evolve by Brownian motion	Yes	Trait conservatism
Dispersal limitation and environmental filtering are the most important processes	No	Include competition based on trait similarity
Community assembly processes operate on both ecological and evolutionary time scales	No	No influence of dispersal on evolutionary patterns
Range centroid and abundance centroid are positively correlated	No	Dispersal from range edge rather than range centroid
Trait-range centroid covariance model sums dispersal limitation and environmental filtering effects	No	Multiplicative model or removal of trait-range centroid covariance
Evolved trait(s) determine species' suitability to examined environmental variables	No	Evolve two traits, environment selects on one, β-diversity measured with other
All species have the same niche breadth and dispersal breadth	?	Niche and/or dispersal breadths vary across species
Environmental niches and dispersal kernals are Gaussian functions	?	More flexible distributions such as the Weibull
Analysis of β-diversity with linear models using distance matrices	No	Nonlinear models, RDA, PCNM

The middle column notes whether or not an assumption can be constrained with empirical data when comparing framework predictions to empirical β-diversity patterns. Examples of alternative assumptions or tools that could be explored in future theoretical studies that modify our framework are noted in the right-hand column.

### Modeling Species Traits and Geographical Ranges through Evolutionary Time

Previous theoretical work has characterized how environmental filtering and competition influence patterns of taxonomic, phylogenetic and functional diversity within local sites [51], [52], [53]. However, within-site diversity does not consider spatial turnover in community composition (i.e. β-diversity). To generate expected β-diversity patterns it is necessary to build from previous work by explicitly considering the spatial distribution of species and environmental conditions at local sites while also addressing the influence of dispersal limitation.

To generate expected taxonomic, phylogenetic and functional β-diversity patterns our model evolves species environmental optima [traits] on a regional phylogeny while tracking species spatial movements. This approach allows local community assembly to be modeled by applying ecological processes to species' trait values and spatial locations, which emerged through evolutionary time. In defining the relationship between species' trait values and their spatial locations we recognize that the processes influencing local community assembly in ecological time must also influence the correlation between species' trait values and their spatial locations in evolutionary time (Fig. 2). If dispersal limitation is strong, species near each other will be closely related and will thus have similar trait values. Regardless of dispersal limitation, if environmental filtering is strong and the environment is spatially structured, species near each other will have similar trait values even if they are not closely related. On the other hand, if the environment is not spatially structured, only dispersal limitation can lead to a correlation between species' trait values and their spatial positions. Therefore, a key assumption of our model is that as species move across the landscape through evolutionary time, the degree to which their trait values correlate with their spatial locations is determined by three factors: the strength of dispersal limitation, the strength of environmental filtering and the degree of environmental spatial structure.

Here, we analytically link dispersal limitation, environmental filtering and the degree of environmental spatial structure to the covariance between species' traits and their spatial positions [range centroids]. To make all variables comparable we assume they are normalized as *z*-scores, which also constrains covariance to range from zero to one. An additive model combining the influences of dispersal limitation and environmental filtering over the covariance between species traits (*T*) and their range centroids (*R*) can be written as
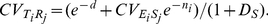
(5)Here 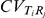 is the species-level covariance between the value of trait *i* and the range centroid position along spatial dimension *j*, and 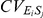 is the community-level covariance between environmental variable *i*, which can select on trait *i*, and spatial dimension *j*. The parameter *n_i_* describes the strength of environmental filtering relative to environmental axis *i*, and is the variance of the Gaussian function describing a species' performance across environmental conditions. As *n_i_* increases, species have broader niches and thus environmental filtering is weaker with respect to axis *i*. Similarly, parameter *d* defines the strength of dispersal limitation and is the variance of the Gaussian dispersal kernel. Dispersal limitation is assumed to be equal across all species and all spatial axes. While *n_i_* values need not be equal with respect to different environmental axes, here we assume that *n*_1_ = *n*_2_. We also make the simplifying assumption that a single value of *n_i_* usefully characterizes the overall degree of environmental filtering across all species in the system. A potential extension of our framework is to relax this assumption by allowing *n_i_* to vary across species, but the chief aim of this research is to characterize the overall or average strength of the process across the species and sites considered. In order to maintain the summed covariance across all traits and dimensions between zero and one, the denominator in Eq. (5) incorporates the number of spatial dimensions (*D_S_*). In turn, if environment and space are normalized as *z*-scores, 
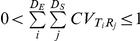
, where *D_E_* is the number of environmental dimensions.

In Eq. (5) broader species' niche functions (weaker environmental filtering) and broader species' dispersal kernels (decreased dispersal limitation) lead to declines in the covariance between trait values and range centroids (i.e. smaller values of 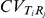). Likewise, narrower niches and dispersal kernels increase the match between a species intrinsic optimal environment (its trait value) and the environmental conditions at the range centroid, where it has maximum abundance (cf. Fig. 2C,D). Note that in simulations underlying Figures 2C and 2D the space-environment covariance was high (≈0.95) such that normalized environmental conditions and spatial positions can effectively be used interchangeably (i.e. x-axes in Figs. 2C,D indicate range centroid spatial position *and* the environmental conditions where abundance is maximized).

The contribution of dispersal limitation to 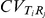 is given by 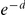 whereby extreme dispersal limitation results in species' traits being correlated with their spatial locations. In this case 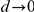 and 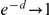, while at the other extreme where dispersal is unlimited 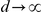 and 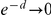. The term 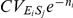 describes the contribution of environmental filtering to 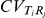. Note that the model assumes that trait *i* is only influenced by environmental dimension *i* such that trait and environment dimensionality must be equal, but need not be the same as the dimensionality of space. This is an assumption that could be relaxed in future versions of the model. When species' niches are very narrow and therefore filtering is very strong, the contribution of filtering to 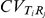 should be equal to 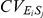. That is, the overall contribution of niche breadth to the trait-range centroid covariance is limited by the degree of spatial structure in environmental variables. As niches become broader and environmental filtering weakens, the contribution of filtering is therefore modeled as a declining fraction of 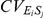, which is itself an empirical input parameter.

While Eq. (5) can be extended to any number of dimensions, we examine a two-dimensional case with two spatial and two environmental axes. As such, species have two environmental optima, one for each abiotic variable, and their range centroid is located in two spatial dimensions. The full covariance matrix defined by Eq. (5) for this two-dimensional case is provided in Table 2, which is the input covariance matrix to the R function ‘sim.char’ (R package *geiger*). Although covariance values are analytically defined, when species' traits are evolved not all realized covariance values will equal their idealized values.

**Table 2:** Idealized trait-space covariance (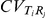) matrix for the evolution of species environmental optima (Trait) and range centroids (Space).

	Trait 1	Trait 2	Space 1	Space 2
Trait 1	1	0	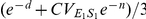	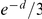
Trait 2	0	1	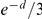	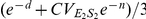
Space 1	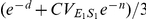	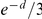	1	0
Space 2	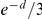	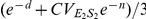	0	1

Species environmental optima and range centroid are both arrayed along two dimensions.

Equation (5) is necessary because the influences of dispersal limitation and environmental filtering over community assembly should be consistent with how those processes structure species' traits and geographic ranges over evolutionary time. If environmental filtering is strong, species' traits will be correlated through evolutionary time with their spatial locations, but only to the degree that the environment is spatially structured (Fig. 2). Likewise, we began with the assumption that the contemporary ability of species to move across the landscape has been maintained through evolutionary time. As a result, Eq. (5) explicitly links the processes influencing the evolutionary-time-scale correlation between trait evolution and range position with the ecological processes governing the assembly of local communities. While other formulations are possible for Eq. (5) (e.g. differentially weighting the relative influence of dispersal limitation and environmental filtering), we have chosen what we believe to be the simplest form that captures the qualitative behavior described above.

### Generating Expectations: Simulation and Analysis Details

In order to demonstrate the use of our framework, provide an example of the expectations it generates, and test its utility in an idealized case, we ran the model with hypothetical, but empirically realistic parameter values. The following procedure was followed for each replicate simulation:

1. Define *n* (equal for all *i*) and *d*. Across simulations *n* and *d* were varied to produce conditions ranging from extreme dispersal limitation and environmental filtering to unlimited dispersal and no filtering. All combinations of eleven logarithmically spaced values of *n* and *d* were evaluated, ranging from 0.0001 to 10.
2. Define spatial positions and environmental conditions of 20 local communities such that 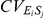
≈0.7 when *i* = *j* and 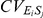
≈0 when *i*≠*j*. This implies that the two environmental axes are independent in our two-dimensional scenario and that each only correlates with one spatial dimension. Setting 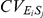
≈0.7 reflects a space-environment R^2^ value near 0.5, which is an empirically relevant degree of environmental spatial structure: most environmental variables are spatially structured at geographic scales (i.e., 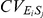
≫0). At extreme levels of spatial autocorrelation (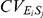
≈1) variance partitioning will have little power to distinguish the unique contributions of environment or space.
3. Generate a regional phylogeny with 500 species using the function ‘birthdeath.tree’ (R package *geiger*) with a birth rate of 0.1 and a death rate of zero, and define the variance-covariance matrix following the equations in Table 2. In turn, via Brownian motion, species' traits were evolved and species' range centroids were tracked along the phylogeny using the variance-covariance matrix as the input to the function ‘sim.char’ (R package *geiger*).
4. Assign species' global relative abundances by randomly drawing from a 500 species log-normal species abundance distribution without replacement.
5. Assemble local communities in the context of the defined *n* and *d* values. For this step one can either sample a fixed number of individuals or sample until a fixed number of species is reached, but both cannot be constrained simultaneously. We assume that limiting resource supply is similar among communities so that each community can support the same number of individuals. In this case realized species richness emerges from the combined influences of assembly processes, the number of individuals in a local community, and the size of the regional species pool. Each local community was assembled by drawing 10,000 individuals with replacement from the regional species pool. For each draw all 500 species were assigned a probability of being included into the assembling community, defined as the product of (*i*) a probability proportional to the species' global relative abundance, (*ii*) a probability from the Gaussian dispersal kernel based on the Euclidean spatial distance between the community's spatial position and a species' range centroid, and (*iii*) a probability from the Gaussian niche function based on the Euclidean environmental distance between the community's environment and a species' environmental optimum (Fig. 1). All 20 communities were assembled independently.
6. Quantify β-diversity for all pairwise community comparisons using Eqs. (1–4). Variance in β-diversity was then partitioned into fractions explained only by spatial distances among communities, only by environmental distances among communities, by spatially structured environmental distances, and a fraction that was unexplained [50]. In addition, the rate of change in β-diversity with spatial and environmental distances was estimated with a multiple regression simultaneously relating β-diversity to spatial and environmental distances. Mean values for each of the four variance compartments and both of the slope estimates were taken across 100 replicates for each of the 121 combinations of *n* and *d* values. These mean values were interpolated (using ‘interp’ in R package *akima*) to generate continuous contour surfaces. The contour surfaces were subsequently used to examine patterns of partitioned variance across the ‘process space’ defined by environmental filtering and dispersal limitation. Regression on distance matrices is used because this is a common approach in empirical β-diversity studies, including recent phylogenetic β-diversity analyses [24]. In future extensions of our framework it would potentially be useful to examine additional statistical tools (e.g. distance-based redundancy analysis) but there is no obvious practical advantage of one approach over all others [34], [54].

## Results and Discussion

### Comparing Intuition-Based and Model-Generated Expectations

**Figure 3:**
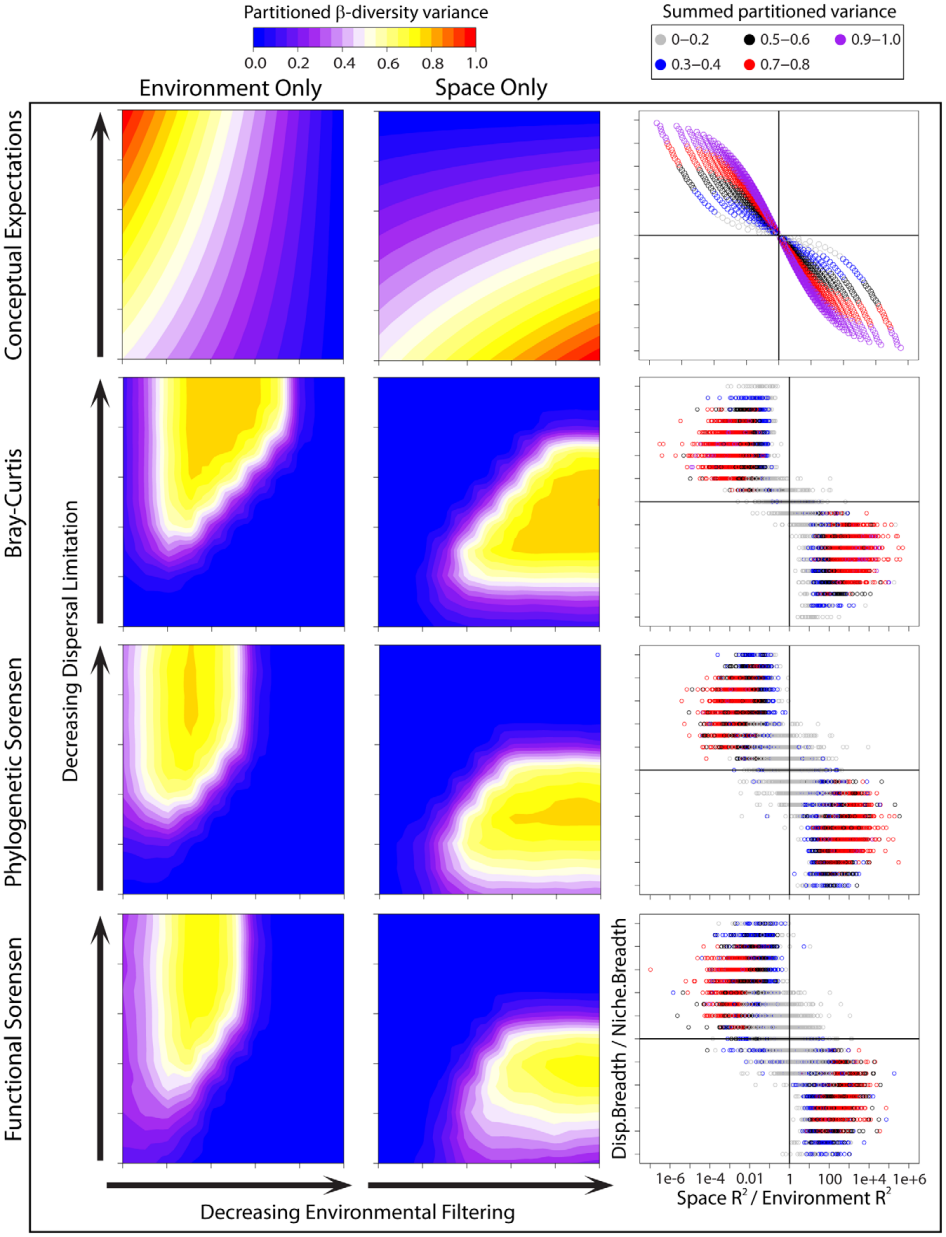
Patterns of variance partitioning across combinations of assembly processes. (*Left 2 columns*) Interpolated variance partitioning across eleven values each of niche breadth (increasing from left to right on each x-axis) and dispersal breadth (increasing from bottom to top on each y-axis) for three β-diversity metrics. The left column is variance partitioned only to the environment, the right column is variance partitioned only to space. Larger niche breadth results in weaker environmental filtering, and a larger dispersal breadth results in weaker dispersal limitation. The intuitive expectation is that variance partitioned to only the environment should decrease moving from the left to the right within each panel in the left column, and variance partitioned only to space should decrease from the bottom to the top within each panel in the right column. Colors in all panels are scaled the same and both axes are log_10_-scale. (*Far right column*) Across all replicate simulations, the ratio of dispersal breadth to niche breadth is plotted against the ratio of variance partitioned to space only and variance partitioned to environment only. Both axes are log_10_-scale. Solid black lines indicate ratios of one. Points are color-coded by the summed variance explained individually by space and environment. Each panel includes data across the 100 replicate simulations for each combination of dispersal and niche breadths. Results using phylogenetic or functional *NN* were qualitatively similar to those using phylogenetic or functional *SOR* and are not shown. See Fig. S1 for phylogenetic and functional *PW* and the space-or-environment ‘shared’ component of partitioned variance.

It is commonly assumed that stronger dispersal limitation leads to greater variance partitioned to space and to steeper slopes (‘spatial slopes’) for the regression of β-diversity against the spatial distance between communities. Likewise, it is often assumed that variance partitioned to the environment should be greater and ‘environmental slopes’ should be steeper when environmental filtering is stronger (e.g., [7], [29]). These conceptual or ‘intuition-based’ expectations are summarized in the top panels of Fig. 3. Comparing the model-generated expectations to the intuition-based expectations showed that no metric or type of β-diversity duplicated these patterns (Figs. 3, S1, S2). In particular, the model predicts that partitioned variances and slope estimates will commonly change as a unimodal function of process strength, and this held under much weaker (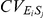
≈0.3) and much stronger (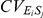
≈0.95) environmental spatial structure (cf. Figs. S1, S3, S4). For example, the variance in phylogenetic *SOR* partitioned to the environment first increased and then decreased going from very weak to very strong environmental filtering. One exception was the variance in functional *PW* partitioned to the environment, which very nearly increased monotonically with increasing environmental filtering (Fig. S1). This is likely due to the environment directly selecting on the traits with which functional β-diversity was calculated.

There are two primary reasons why explained variances and slope values increase and then decrease as a given process gets stronger. First, as the strength of either dispersal limitation or environmental filtering increases, species richness decreases (Fig. S5). When there are few species within each local community, it is unlikely that any two communities will share many species regardless of spatial or environmental distance. Second, when processes are extremely strong there will be complete turnover even over relatively short spatial or environmental distances. In turn, similarity values go to zero across the majority of observed spatial and environmental distances. Fitting statistical functions to these sorts of data results in a poor fit (thus low explained variance) because most data have the same value and the only variation in the data is over a small range of short distances. Shallow slopes are also expected in this case because the majority of data fall on the zero-similarity line so that there is no decay in similarity across most of the range in spatial or environmental distances (Fig. S6). In such extreme cases it would not be reasonable to infer that processes are weak simply due to low explained variance. Most empirical systems are not as extreme as the example provided in Fig. S6B and are characterized by at least moderately low partitioned variance (>10%) [29], in which case the appropriate inference becomes ambiguous. Unimodal variance partitioning patterns must therefore be taken into consideration when making inferences for most empirical systems.

An important implication of unimodal patterns in explained variance and slope estimates is that empirical observations of low explained variance and shallow slopes do not necessarily indicate weak dispersal limitation and environmental filtering. It may, however, be possible to use relative magnitudes of partitioned variances and slopes to infer the relative influences of dispersal limitation and environmental filtering. The degree to which this is useful can be examined by plotting the ratio of process strengths (i.e. *d*/*n*) against the ratio of partitioned variances or the ratio of fitted slopes for each simulated set of communities. The expected relationship is provided in the upper right panel of Fig. 3: when variance partitioned to space is larger than variance partitioned to the environment, one intuitively expects that dispersal limitation is stronger than environmental filtering, and vice versa. The same is true for slope estimates. As such, all data points are expected to fall in the upper left and lower right quadrants of the right-hand panels in Fig. 3. If a substantial number of simulations fall into the lower left or upper right quadrants, using intuition alone would lead to the wrong inference regarding the relative influences of dispersal limitation and environmental filtering.

For all metrics and all three types of β-diversity a large fraction of model-generated expectations fell into the upper left or lower right quadrants (Figs. 3, S1, S2, right column). Across all 121 combinations of *d* and *n* the percentage of replicate simulations that were consistent with the intuitive expectations based on variance partitioning ratios were 86% for *BC*, 82% for taxonomic *SOR*, 76% for phylogenetic *PW*, 82% for phylogenetic *SOR*, 75% for functional *PW*, and 81% for functional *SOR*. Similar patterns were found under different degrees of environmental spatial structure and for the ratio of the spatial slope to the environmental slope. As such, the variance partitioning ratio can be a useful indicator of which process, dispersal limitation or environmental filtering, is more influential in community assembly. Caution is warranted, however, when total explained variation is low (<20%) or when the environment has strong spatial structure (cf. Figs. S1, S3, S4, right columns).

The departures between intuition-based and model-generated expectations clearly show that interpreting β-diversity patterns from intuition alone can provide incorrect inferences of underlying processes. A similar conclusion was reached by Smith and Lundholm [34] and Gilbert and Bennett [54] for taxonomic β-diversity, and we show that the same conclusion holds for phylogenetic and functional β-diversity. It is worth noting that, with respect to taxonomic β-diversity, our results are very similar to those of Smith and Lundholm [34] even though our simulation model is entirely different. This suggests that the presence of non-monotonic functions relating explained variance to the strength of underlying processes is likely the rule rather than the exception. Inferring community assembly processes therefore requires that empirical patterns be compared to expectations generated by process-based simulation frameworks such as the one derived here (for a similar perspective see [38], [39], [40], [41]).

### Testing the Framework and Merging Taxonomic, Phylogenetic and Functional β-Diversity

We have shown that interpreting patterns of taxonomic, phylogenetic or functional β-diversity using intuition alone can lead to misleading inferences regarding both the relative and absolute strengths of dispersal limitation and environmental filtering. The key question now is if empirical patterns are compared to model-generated expectations, do the resulting inferences closely estimate the true strengths of underlying processes? To answer this question and test our framework we next define how strong dispersal limitation and environmental filtering are, use our framework to generate β-diversity patterns, and then determine whether or not those β-diversity patterns can be used to go ‘backwards’ to unambiguously and correctly infer the known process strengths that were used in the simulation. It must be recognized that this is a necessary step, but only a first step in testing our framework, and represents the best-case scenario. If the framework does not pass this simple test, it must be modified. We also use this test as an opportunity to provide an example of how empiricists can couple our framework to specific empirical systems.

To challenge the framework as much as possible we chose a location in process space where there was a strong departure between the intuition-based expectations and the model-generated expectations. That location was the lower left corner of process space corresponding to extreme dispersal limitation and environmental filtering (*d* = *n* = 0.0001). We selected one set of local communities simulated under these conditions to serve as example ‘empirical’ data. For taxonomic and phylogenetic β-diversity, variation partitioned uniquely to either space or the environment in this set of communities was less than 0.038 (Fig. 3). If these were actual empirical results, the intuitive interpretation would be that taxonomic and phylogenetic community structure are not influenced by either dispersal limitation or environmental filtering. The fractions of functional *PW* partitioned to space and to the environment were 0.014 and 0.31, respectively, which would naively lead one to infer that functional composition is primarily governed by environmental filtering. Neither of these inferences would be correct.

Rather than relying on intuition, the simulations provide a framework for what combinations of process strengths would be expected to yield the empirically observed variance partitioning results. As a first step, we can select the regions of process space where the model-generated variance partitioning values closely match the ‘empirical’ values. If these regions are large, inferences of process strengths will be ambiguous, indicating that different process strengths lead to the same β-diversity patterns.

**Figure 4:**
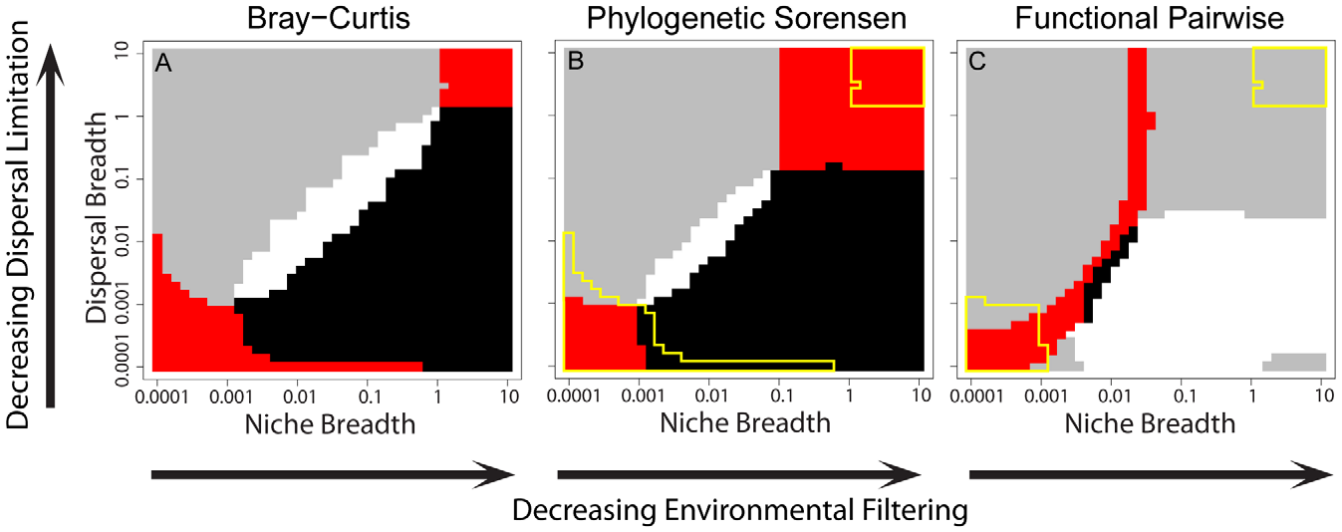
Example of using ‘empirical’ (see text) analyses of β-diversity to infer community assembly processes. For each β-diversity metric, empirical variance partitioning results are first compared to model-based expectations. The regions of process space where model expectations closely match empirical results for variance partitioned to space (grey) or the environment (black) are shown. Regions where model expectations are consistent with both the space and environmental variance partitioning results are highlighted in red. (A) Results for *BC*; (B) Results for phylogenetic *SOR*, where yellow delineates regions of overlap in panel A. (C) Results for functional *PW*, where yellow delineates the intersection of red and yellow regions in panel B. True values of niche (*n*) and dispersal breadths (*d*) must reside where yellow and red intersect in panel C. The actual parameter values in this test case were *n* = *d* = 0.0001, consistent with the inference provided by combining taxonomic, phylogenetic and functional β-diversity.

In the case of *BC*, variance partitioned to space was 0.012 which, after including some error around this value, corresponds to the swath of process space indicated by the grey and red regions in Fig. 4A (cf. the blue region of the ‘space only’ Bray-Curtis plot in Fig. 3). Variance partitioned to the environment was 0.005, which corresponds to the black and red regions of Fig. 4A (cf. the blue region of the ‘environment only’ Bray-Curtis plot in Fig. 3). Only the combinations of dispersal limitation and environmental filtering highlighted by the red regions of Fig. 4A would yield variance partitioning results similar to what was observed empirically based on the *BC* β-diversity measure.

Using *BC* alone results in two plausible regions of process space that could have resulted in the observed patterns–either both processes are very strong or both are very weak. Using the framework with *BC* alone has therefore provided an ambiguous answer regarding the strength of assembly processes. Using variance partitioning based on phylogenetic *SOR* did not greatly reduce this ambiguity: the upper right and lower left corners of process space were consistent with the ‘empirical’ phylogenetic *SOR* variance partitioning results (red regions, Fig. 4B). Variance partitioning based on functional *PW* again highlights multiple regions where the ‘empirical’ data were consistent with the theoretical expectations. However, these regions of consistency start from the lower left corner and sweep up through the central region of process space (Fig. 4C). The only region that was consistent with the variance partitioning results of all three types of β-diversity was a small area in the lower left corner (Fig. 4C). Therefore, while each type of β-diversity provided ambiguous inferences when considered alone, combining patterns from all three types of β-diversity resulted in an unambiguous inference that closely estimated the known process strengths (*d* = *n* = 0.0001) with a reasonably small degree of error.

This first-order test of our framework again shows that intuitive interpretations of β-diversity patterns can frequently lead to incorrect inferences. This is true even if taxonomic, phylogenetic and functional β-diversity are examined together. Results from the test also suggest that our framework is a useful tool for moving beyond intuition-based interpretations, and that by using the framework in conjunction with real empirical patterns of taxonomic, phylogenetic and functional β-diversity one can rigorously infer the absolute strengths of dispersal limitation and environmental filtering. One component of the variance partitioning analyses that we did not utilize is the space-or-environment ‘shared’ component. We have not used the shared component because it cannot be uniquely ascribed to either space or the environment, it has no clear intuition-based expectations, and its model-generated expectations are heavily influenced by the degree of environmental spatial structure (cf. Figs. S1, S3, S4). Nonetheless, the shared component may be useful in some cases and we encourage using it alongside the variance components ascribed uniquely to space and to the environment. In fact there seems to be little cost in using all three variance components simultaneously: doing so resulted in estimated process strengths very close to the known process strengths (*d* = *n* = 0.0001) across a broad range of environmental spatial structure (Figs. S7, S8, and S9).

### Extensions and Caveats

All tests of our framework indicate that it provides rigorous, quantitative estimates for the strengths of dispersal limitation and environmental filtering. Most directly, these estimates are variances of a Gaussian dispersal kernel and a Gaussian niche function. A powerful aspect of our framework is that when coupled to an empirical system, these Gaussian curves have units set by that system, and thus provide an opportunity to test the model's predictions. The dispersal function is a quantitative description of how the probability of dispersal declines as distance from a reproducing individual increases. The niche function is a quantitative description of how survival probability declines as the environment increasingly deviates from a species' optimal environment. In systems where it is feasible, the estimated functions could be compared to field estimated dispersal kernels and experimental estimates of organismal performance across environmental axes of interest. Testing the framework in this way would increase understanding of the framework's capabilities and limitations, and in turn, provide refined guidance on how to best apply the framework to empirical systems.

We have outlined a straightforward procedure for inferring community assembly processes by combining analyses of taxonomic, phylogenetic and functional β-diversity. We look forward to empiricists using frameworks like that developed here, and an ensuing discussion on how to improve upon these types of theoretical tools. One limitation of any analysis relating community structure to environmental variables is that interpretations are limited to the influence of measured aspects of the environment. This is true for analyses done in the context of our framework as well. We recommend that empiricists carefully choose environmental variables and first examine their influence one by one within a theoretical framework. If multiple variables are found to be important, they can be combined in a framework like that developed here to look for interactive effects. Regardless of the outcome, important information will be gained regarding the influence of specific environmental variables. We further emphasize that our framework provides interpretations for processes that influence whole community structure. If some environmental variable influences a small subset of species, this should be reflected in our framework as a relatively weak influence of environmental filtering with respect to that environmental variable. One should also note that in cases where only taxonomic data are available, a framework like that developed here is still essential. In the example we used to test our framework, an empiricist with only taxonomic data would realize that their data are consistent with two very different regions of process space and would refrain from making an intuitive but unjustified conclusion. On the other hand, in less extreme situations taxonomic data may be all that is necessary to correctly infer process strengths. An empiricist cannot know this, however, without comparing empirical patterns to theoretical expectations like those generated here.

It is important to recognize that the framework developed here is only one of many possibilities (e.g., [34]), and that a small portion of the realistic combinations of potential processes, scenarios and parameters has been explored. As more studies combine taxonomic, phylogenetic and functional β-diversity, alternative and updated frameworks should be developed. For example, one could include a competitive process during community assembly (e.g., as in [53]). We encourage these developments and recommend that explicit comparisons be made among frameworks so that we have a clear understanding of the tradeoffs inherent in using one approach over another. To facilitate further developments we summarize, in Table 1, major assumptions of our framework that can be modified in future theoretical efforts and in studies that link frameworks like that developed here to specific empirical systems. This sort of systematic approach will help minimize the disagreements and misunderstandings that were common as null models were developed for community assembly rules (e.g., [37], [55], [56], [57], [58], [59]).

Regardless of the specific approach taken in future studies, any extension must retain five fundamental components: (1) the ability to empirically constrain parameter values; (2) coordinated evolution of species traits and spatial locations; (3) inclusion of explicit effects of spatially structured environments; (4) local community assembly governed by the combined influences of clearly defined processes; and (5) an overall structure that allows empirical patterns to be directly related to the theoretical expectations. We are confident that placing empirical β-diversity patterns in the context of model-generated expectations will lead to a much deeper understanding of community assembly processes and how they vary through space, time and across taxa.

## Supporting Information

Figure S1 **Patterns of variance partitioning across combinations of assembly processes.** (*Left 3 columns*) Interpolated variance partitioning across eleven values each of niche breadth (increasing from left to right on each x-axis) and dispersal breadth (increasing from bottom to top on each y-axis) for five β-diversity metrics, including those presented in Figure 3. The left column is variance partitioned only to the environment, middle column is variance partitioned to space or the environment, and the right column is variance partitioned only to space. Larger niche breadth results in weaker environmental filtering, and larger dispersal breadth results in weaker dispersal limitation. See Figure 3 for intuitive expectations of the ‘space only’ and ‘environment only’ components of partitioned variance, and note that there is no obvious intuitive expectation for patterns of the space-or-environment component. The variance partitioned to the space-or-environment component is intermediate relative to the more extreme levels of environmental spatial structure (see Figs. S3, S4), as expected with the intermediate degree of environmental spatial structure used here (space-environment covariance≈0.7). Colors in all panels are scaled the same and both axes are log_10_-scale. (*Far right column*) Across all replicate simulations, the ratio of dispersal breadth to niche breadth is plotted against the ratio of variance partitioned to space only and variance partitioned to environment only. Both axes are log_10_-scale. Solid black lines indicate ratios of one. Points are color-coded by the summed variance explained individually by space and environment. Each panel includes data across the 100 replicate simulations for each combination of dispersal and niche breadths.
(TIF)

Figure S2 **Patterns of multiple regression slope parameters across combinations of assembly processes.** (*Left 2 columns*) Interpolated multiple regression slope parameters, where vertical and horizontal axes are as in Figure S1. Note that color bars are scaled differently in each panel. More negative slopes indicate higher turnover in community structure at greater environmental (*left column*) or spatial (*right column*) distances. (*Far right column*) The ratio of dispersal breadth to niche breadth plotted against the ratio of the spatial slope to the environmental slope. Both axes are log_10_-scale. Points are color-coded by quantile scores across the distribution of summed spatial and environmental slopes. Note that steeper slopes (indicated by larger quantile scores) generally fall into the upper left and lower right quadrants, thereby correctly identifying the more influential process. See the main text and Figure S1 for additional details.
(TIF)

Figure S3 **Patterns of variance partitioning across combinations of assembly processes.** As in Figure S1, but under stronger environmental spatial structure (space-environment covariance≈0.95, as compared to 0.7). Note that nearly all explained variance is within the space-or-environment component, as expected when space and environment are confounded with each other.
(TIF)

Figure S4 **Patterns of variance partitioning across combinations of assembly processes.** As in Figure S1, but under weaker environmental spatial structure (space-environment covariance≈0.3, as compared to 0.7). Note that nearly all explained variance is within the two unique components, as expected when space and environment are largely independent of each other.
(TIF)

Figure S5 **Species richness patterns across combinations of assembly processes.** Interpolated mean local community species richness across all communities and all replicates for 11 values each of niche breadth and dispersal breadth.
(TIF)

Figure S6 **Example simulation outputs relating similarity in taxonomic composition among local sites to the spatial distances among local sites.** In both panels environmental filtering was set to be very weak (variance of niche function = 10). Dispersal limitation was set to be (A) of moderate strength (variance of dispersal kernal = 10^−1.5^), or (B) very strong (variance of dispersal kernal = 10^−4^). Note that in (A) similarity declines continuously with spatial distance whereas in (B) similarity declines to zero over very short spatial distances. The distribution of data in (A) results in higher explained variance and a steeper distance decay slope, as compare to (B).
(TIF)

Figure S7 **Example of using ‘empirical’ (see text) analyses of β-diversity to infer community assembly processes.** For each β-diversity metric empirical variance partitioning results are first compared to model-based expectations using all three variance partitioning components (‘space only’, ‘environment only’, and ‘space or environment’). The regions of process space where model expectations closely match empirical results for variance partitioned to all three components are shown in red. Environmental spatial structure was intermediate and as in Figure 4 (space-environment covariance≈0.7). (A) Results for *BC*; (B) Results for phylogenetic *SOR*, where blue delineates regions of overlap in panel A. (C) Results for functional *PW*, where blue delineates the intersection of red and blue regions in panel B. True values of niche (*n*) and dispersal breadths (*d*) must reside where blue and red intersect in panel C. The actual parameter values in this test case were *n* = *d* = 0.0001, consistent with the inference provided by combining taxonomic, phylogenetic and functional β-diversity.
(TIF)

Figure S8 **Example of using ‘empirical’ (see text) analyses of β-diversity to infer community assembly processes.** As in Figure S7, but in the case where the environment is strongly spatially structured (space-environment covariance≈0.95).
(TIF)

Figure S9 **Example of using ‘empirical’ (see text) analyses of β-diversity to infer community assembly processes.** As in Figure S7, but in the case where the environment is weakly spatially structured (space-environment covariance≈0.3).
(TIF)
