# Supplementary figures and images for: Inferring Ecological Processes from Taxonomic, Phylogenetic and Functional Trait β-Diversity
Source: PLoS One. 2011 Jun 17;6(6):e20906. doi: 10.1371/journal.pone.0020906 (PMC3117851; doi:10.1371/journal.pone.0020906)

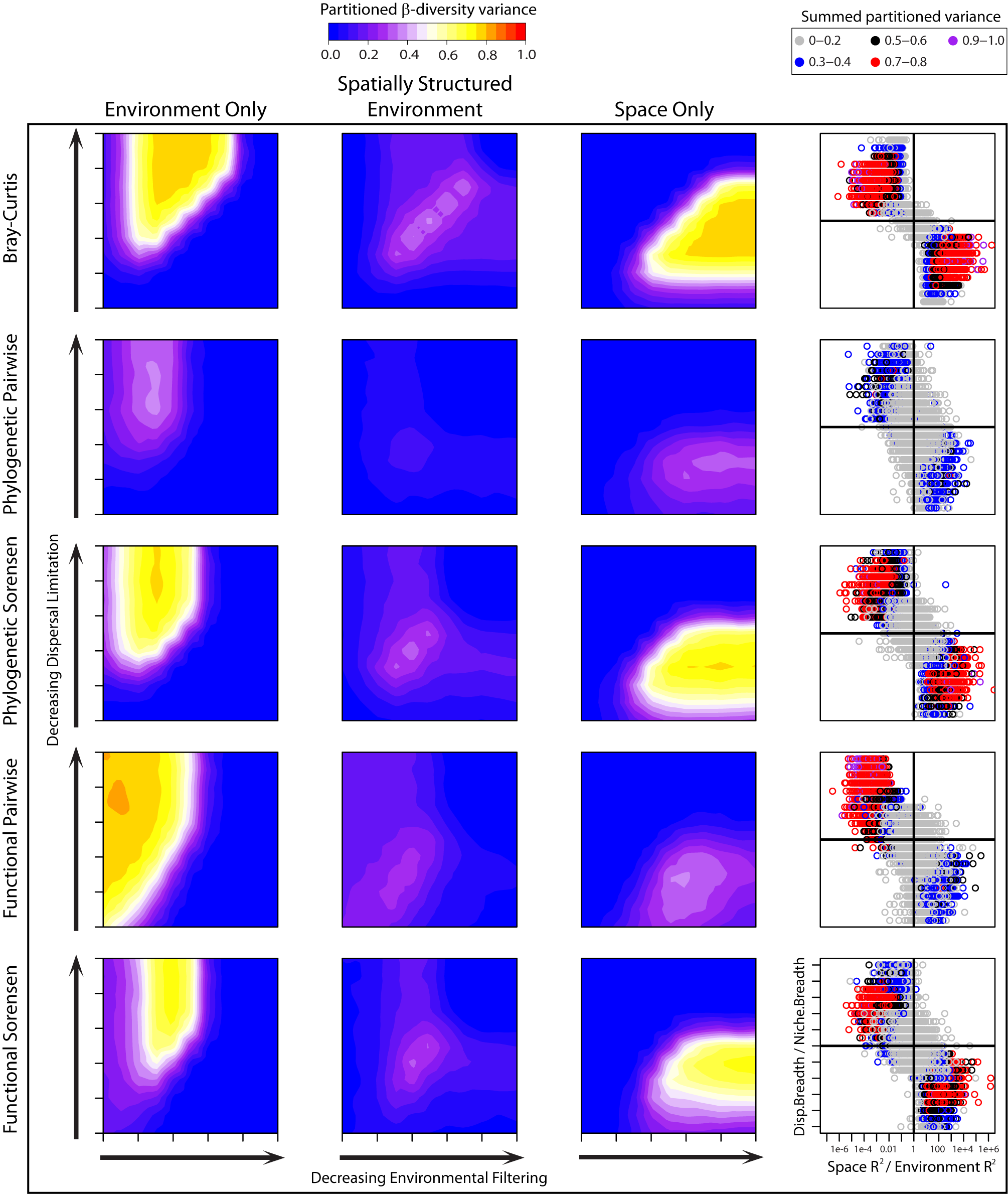

Supplement: Figure S1 — Patterns of variance partitioning across combinations of assembly processes. (Left 3 columns) Interpolated variance partitioning across eleven values each of niche breadth (increasing from left to right on each x-axis) and dispersal breadth (increasing from bottom to top on each y-axis) for five β-diversity metrics, including those presented in Figure 3. The left column is variance partitioned only to the environment, middle column is variance partitioned to space or the environment, and the right column is variance partitioned only to space. Larger niche breadth results in weaker environmental filtering, and larger dispersal breadth results in weaker dispersal limitation. See Figure 3 for intuitive expectations of the ‘space only’ and ‘environment only’ components of partitioned variance, and note that there is no obvious intuitive expectation for patterns of the space-or-environment component. The variance partitioned to the space-or-environment component is intermediate relative to the more extreme levels of environmental spatial structure (see Figs. S3, S4), as expected with the intermediate degree of environmental spatial structure used here (space-environment covariance≈0.7). Colors in all panels are scaled the same and both axes are log10-scale. (Far right column) Across all replicate simulations, the ratio of dispersal breadth to niche breadth is plotted against the ratio of variance partitioned to space only and variance partitioned to environment only. Both axes are log10-scale. Solid black lines indicate ratios of one. Points are color-coded by the summed variance explained individually by space and environment. Each panel includes data across the 100 replicate simulations for each combination of dispersal and niche breadths. (TIF) [file pone.0020906.s001.tif]

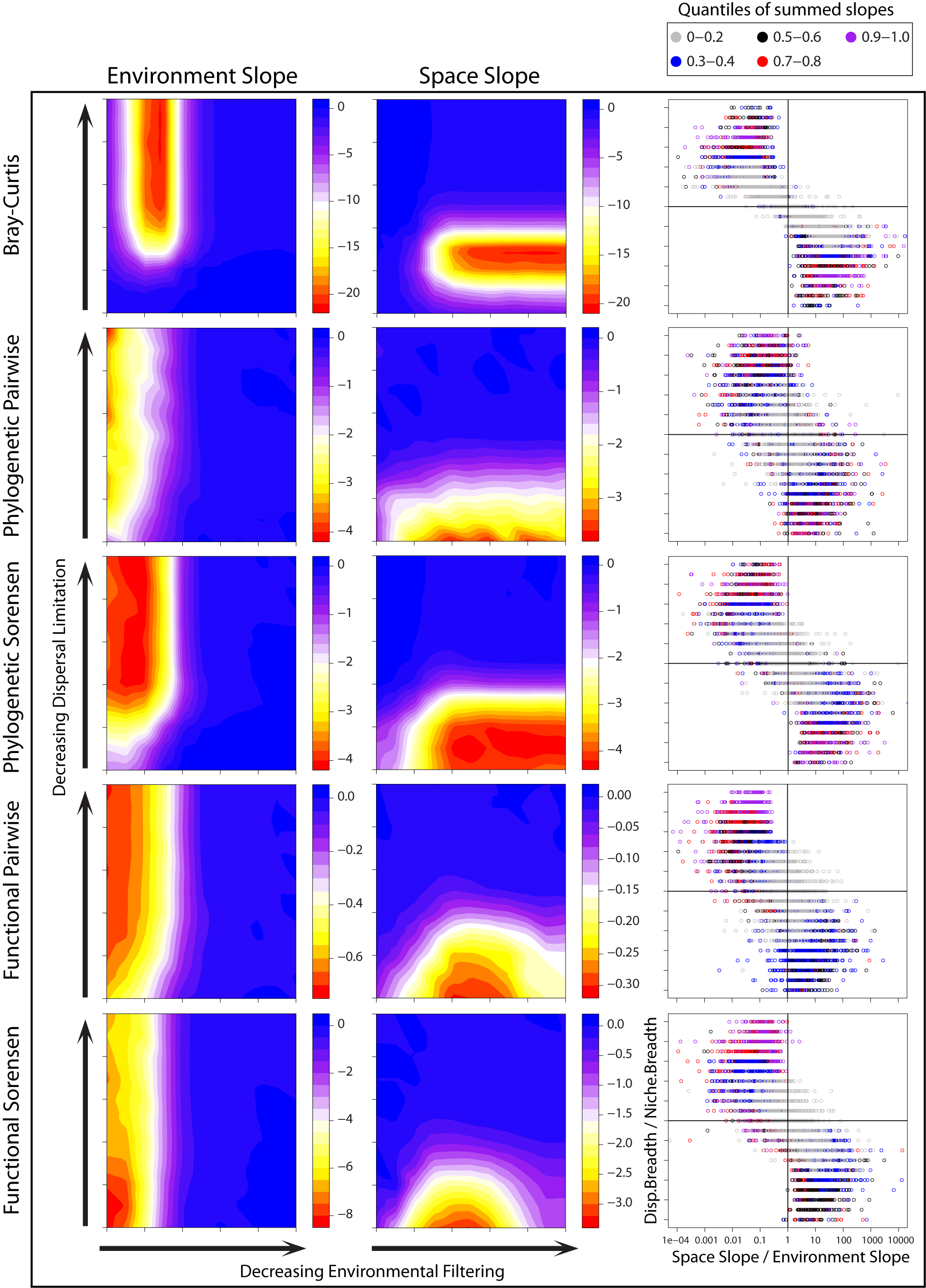

Supplement: Figure S2 — Patterns of multiple regression slope parameters across combinations of assembly processes. (Left 2 columns) Interpolated multiple regression slope parameters, where vertical and horizontal axes are as in Figure S1. Note that color bars are scaled differently in each panel. More negative slopes indicate higher turnover in community structure at greater environmental (left column) or spatial (right column) distances. (Far right column) The ratio of dispersal breadth to niche breadth plotted against the ratio of the spatial slope to the environmental slope. Both axes are log10-scale. Points are color-coded by quantile scores across the distribution of summed spatial and environmental slopes. Note that steeper slopes (indicated by larger quantile scores) generally fall into the upper left and lower right quadrants, thereby correctly identifying the more influential process. See the main text and Figure S1 for additional details. (TIF) [file pone.0020906.s002.tif]

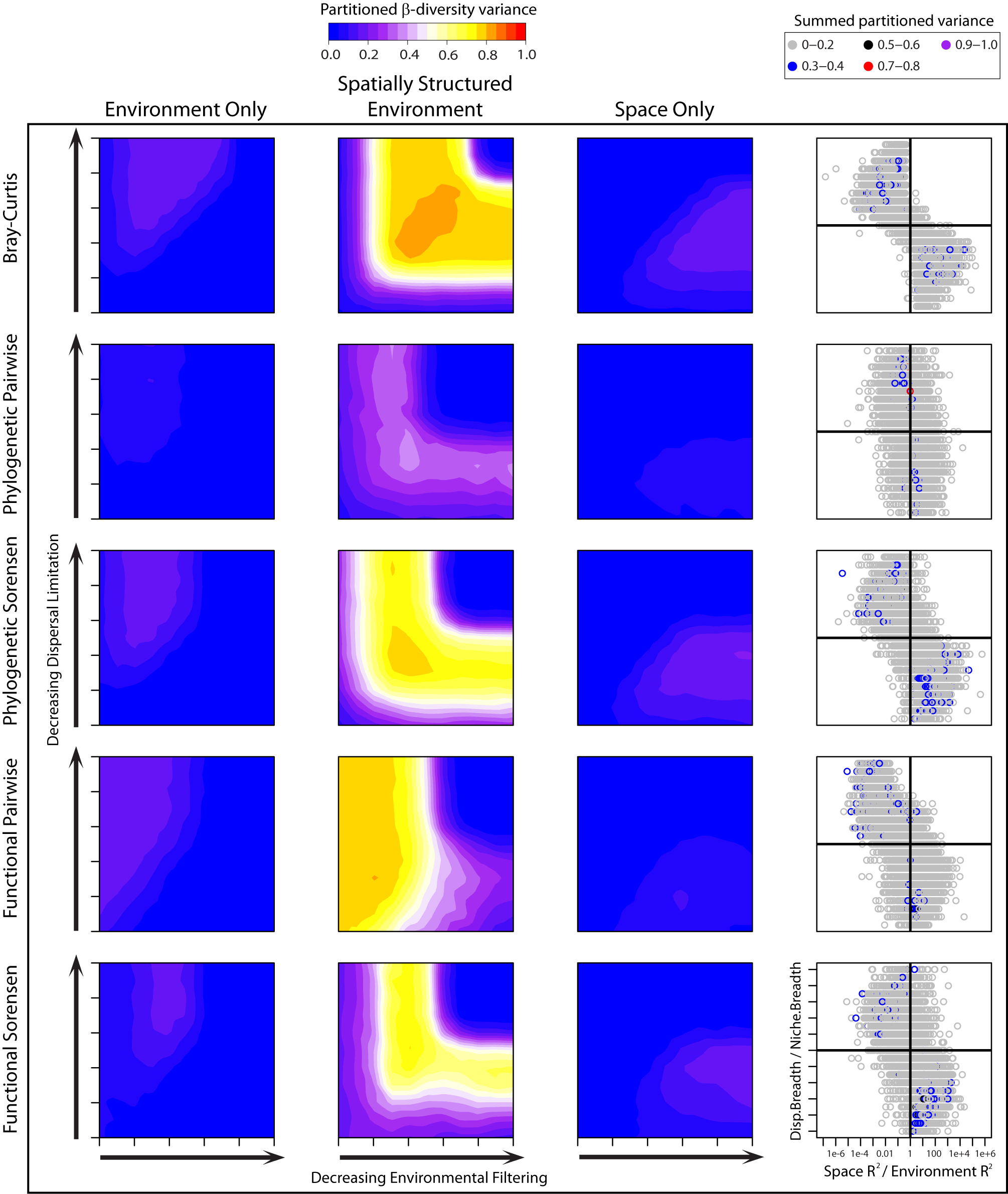

Supplement: Figure S3 — Patterns of variance partitioning across combinations of assembly processes. As in Figure S1, but under stronger environmental spatial structure (space-environment covariance≈0.95, as compared to 0.7). Note that nearly all explained variance is within the space-or-environment component, as expected when space and environment are confounded with each other. (TIF) [file pone.0020906.s003.tif]

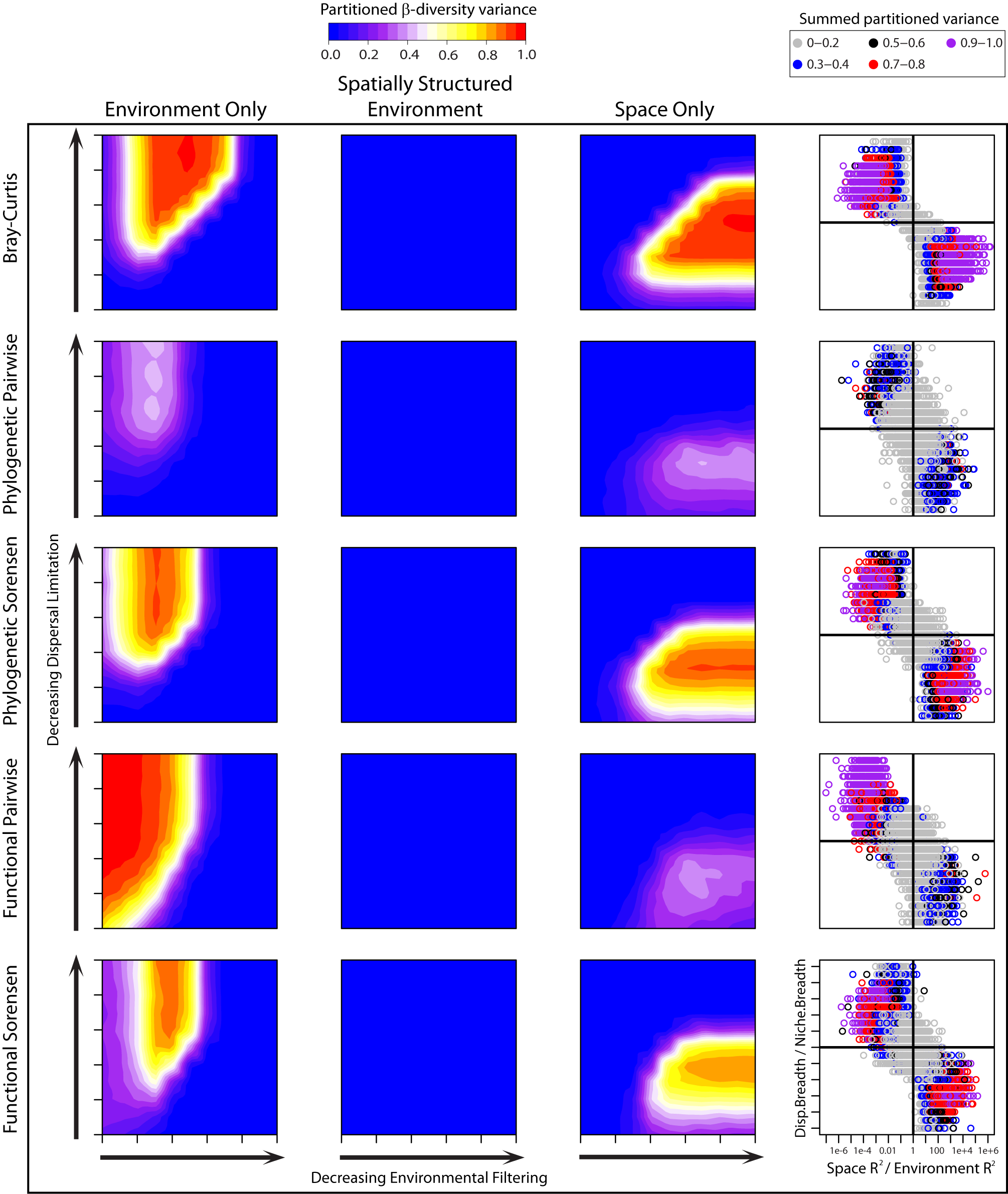

Supplement: Figure S4 — Patterns of variance partitioning across combinations of assembly processes. As in Figure S1, but under weaker environmental spatial structure (space-environment covariance≈0.3, as compared to 0.7). Note that nearly all explained variance is within the two unique components, as expected when space and environment are largely independent of each other. (TIF) [file pone.0020906.s004.tif]

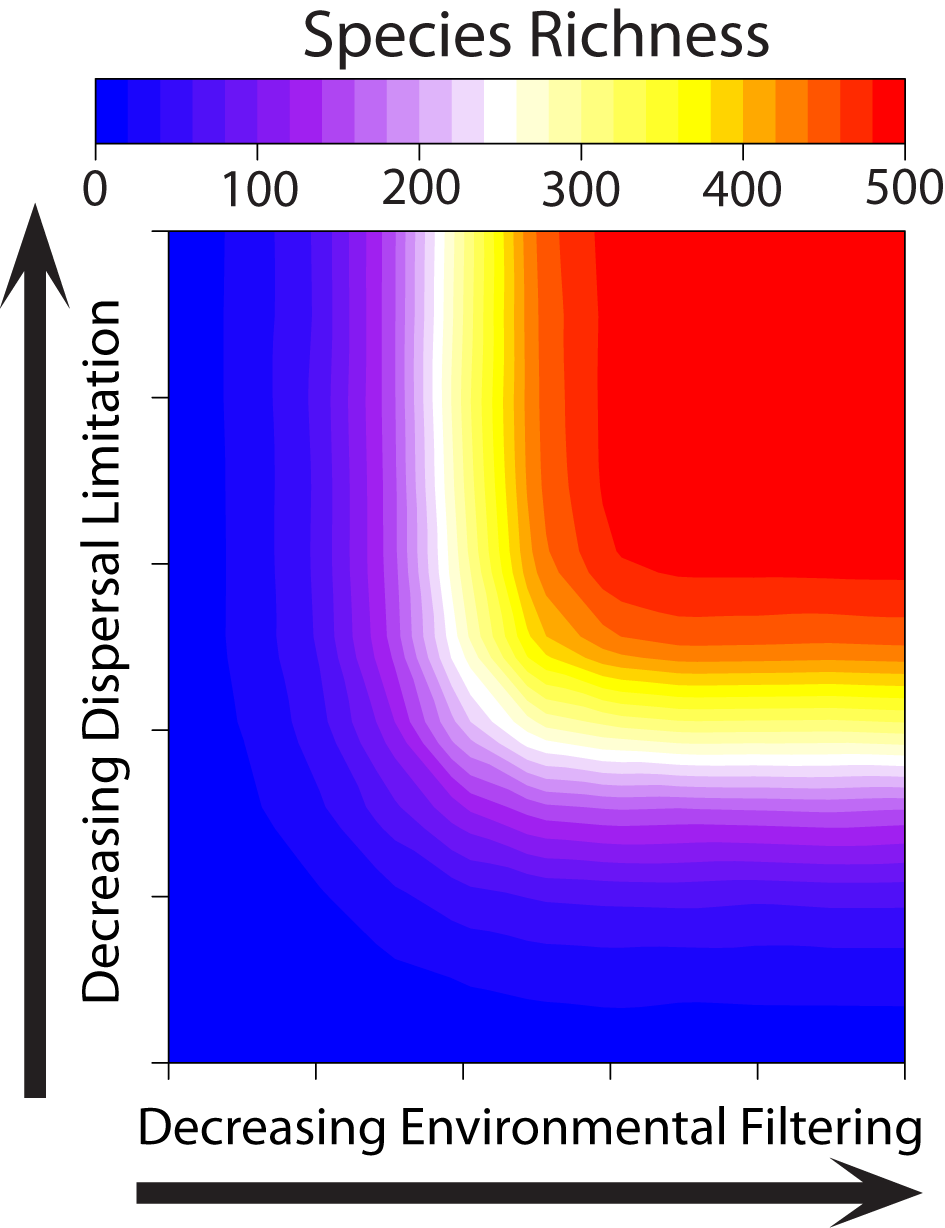

Supplement: Figure S5 — Species richness patterns across combinations of assembly processes. Interpolated mean local community species richness across all communities and all replicates for 11 values each of niche breadth and dispersal breadth. (TIF) [file pone.0020906.s005.tif]

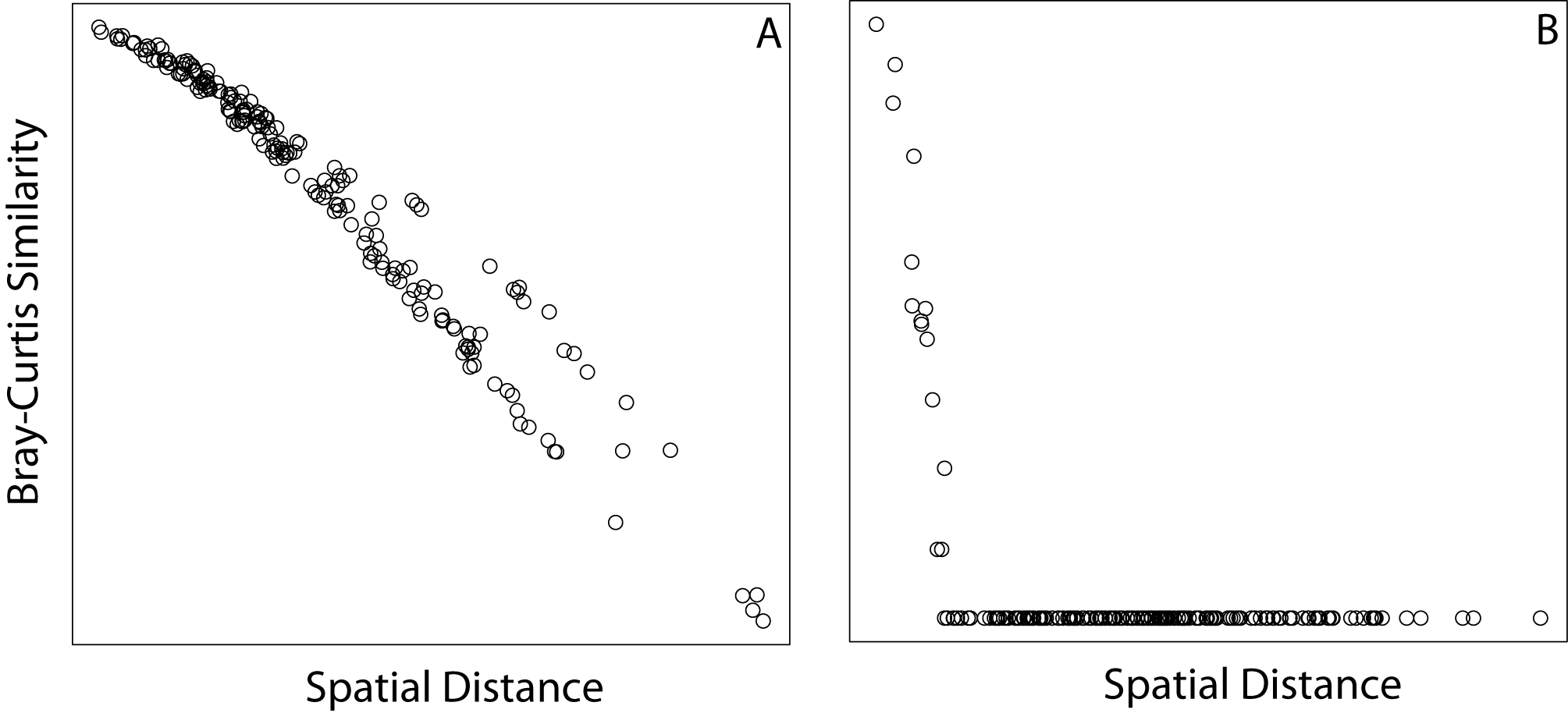

Supplement: Figure S6 — Example simulation outputs relating similarity in taxonomic composition among local sites to the spatial distances among local sites. In both panels environmental filtering was set to be very weak (variance of niche function = 10). Dispersal limitation was set to be (A) of moderate strength (variance of dispersal kernal = 10−1.5), or (B) very strong (variance of dispersal kernal = 10−4). Note that in (A) similarity declines continuously with spatial distance whereas in (B) similarity declines to zero over very short spatial distances. The distribution of data in (A) results in higher explained variance and a steeper distance decay slope, as compare to (B). (TIF) [file pone.0020906.s006.tif]

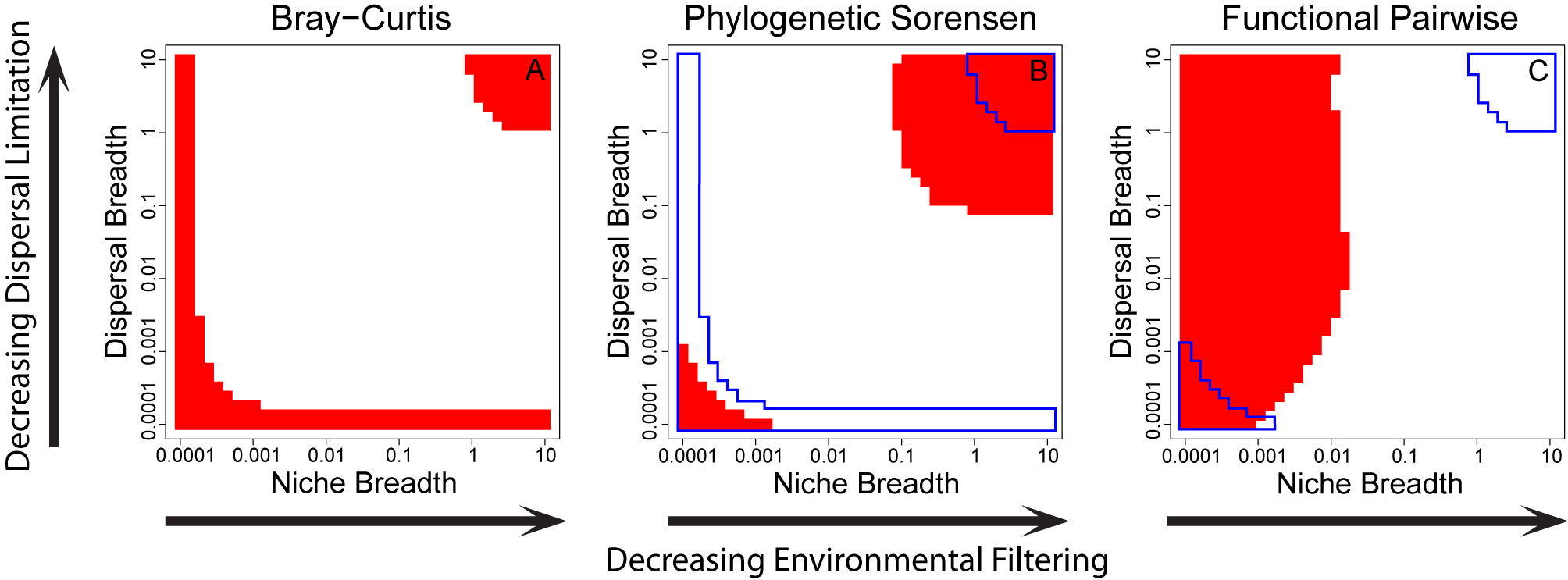

Supplement: Figure S7 — Example of using ‘empirical’ (see text) analyses of β-diversity to infer community assembly processes. For each β-diversity metric empirical variance partitioning results are first compared to model-based expectations using all three variance partitioning components (‘space only’, ‘environment only’, and ‘space or environment’). The regions of process space where model expectations closely match empirical results for variance partitioned to all three components are shown in red. Environmental spatial structure was intermediate and as in Figure 4 (space-environment covariance≈0.7). (A) Results for BC; (B) Results for phylogenetic SOR, where blue delineates regions of overlap in panel A. (C) Results for functional PW, where blue delineates the intersection of red and blue regions in panel B. True values of niche (n) and dispersal breadths (d) must reside where blue and red intersect in panel C. The actual parameter values in this test case were n = d = 0.0001, consistent with the inference provided by combining taxonomic, phylogenetic and functional β-diversity. (TIF) [file pone.0020906.s007.tif]

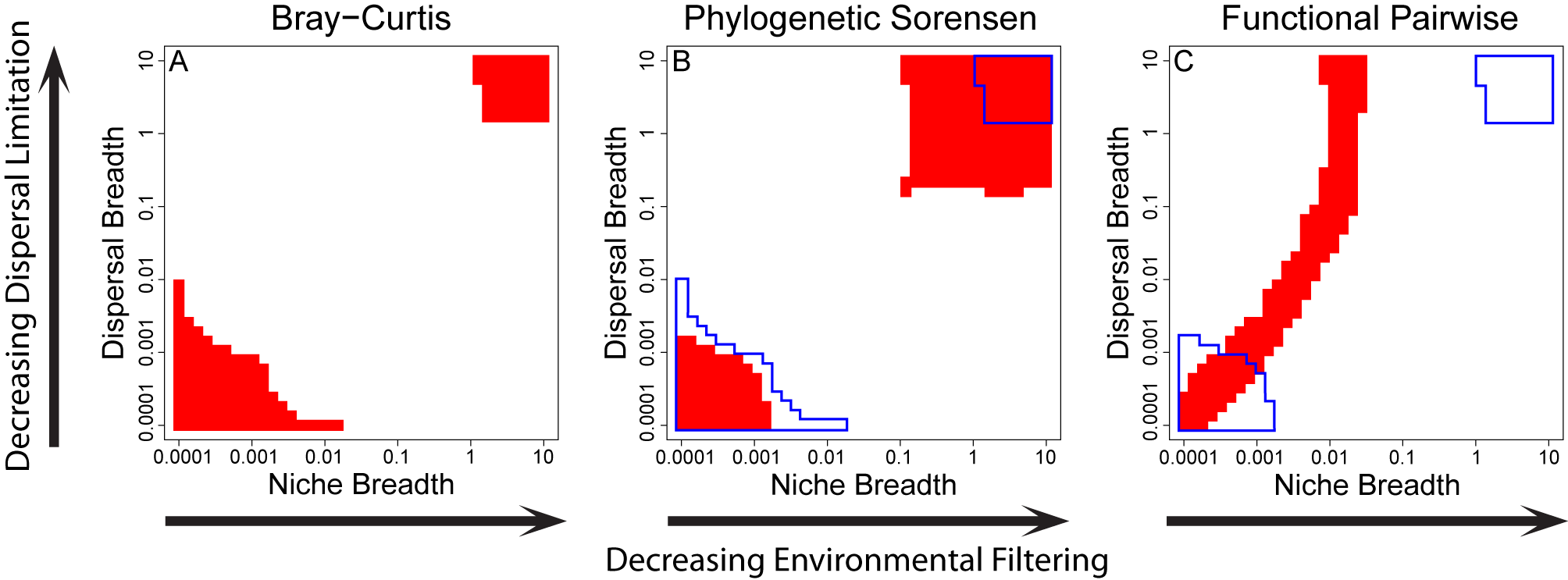

Supplement: Figure S8 — Example of using ‘empirical’ (see text) analyses of β-diversity to infer community assembly processes. As in Figure S7, but in the case where the environment is strongly spatially structured (space-environment covariance≈0.95). (TIF) [file pone.0020906.s008.tif]

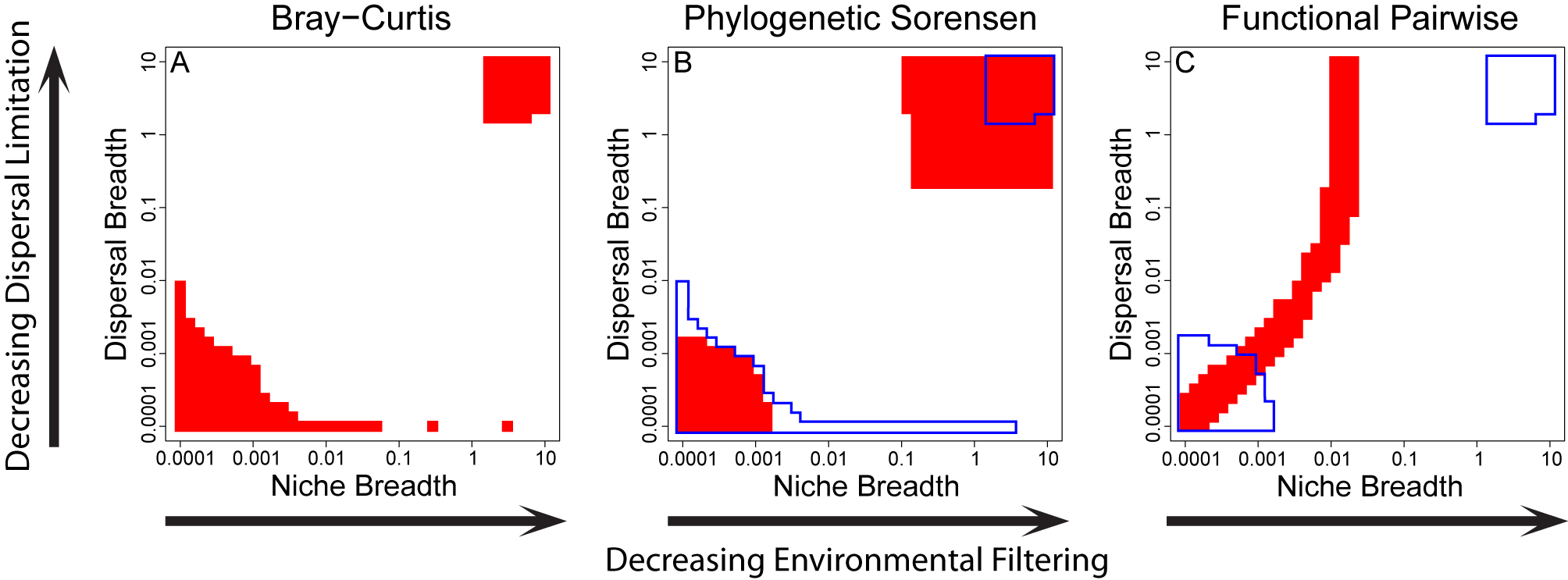

Supplement: Figure S9 — Example of using ‘empirical’ (see text) analyses of β-diversity to infer community assembly processes. As in Figure S7, but in the case where the environment is weakly spatially structured (space-environment covariance≈0.3). (TIF) [file pone.0020906.s009.tif]
